# Supplementary material for: Highly Skin-Conformal Laser-Induced Graphene-Based Human Motion Monitoring Sensor
Source: Nanomaterials (Basel). 2021 Apr 8;11(4):951. doi: 10.3390/nano11040951 (PMC8068237; doi:10.3390/nano11040951)
Supplement: Supplementary file 1 [file nanomaterials-11-00951-s001.zip › nanomaterials-1171200SI/SI.docx]

Supporting Information

Highly Skin-Conformal Laser-Induced Graphene-Based Human Motion Monitoring Sensor

Sung-Yeob Jeong ^1,†^, Jun-Uk Lee ^2,†^, Sung-Moo Hong ^3^, Chan-Woo Lee ^2^, Sung-Hwan Hwang ^2^, Su-Chan Cho and Bo-Sung Shin ^3,^*

^1^ Department of Mechanical Engineering, The University of Tokyo, Tokyo, 113-8656, Japan, ysjsykj8025@naver.com

^2^ Department of Cogno-Mechatronics Engineering, Pusan National University, Pusan, 46241, Korea, lju3534@naver.com(J.-U.L.); cwleeho2@naver.com (C.-W.L.); po78765@naver.com (S.-H.H.); cho_brian@naver.com (S.-C.C.);

^3^ Interdisciplinary Department for Advanced Innovative Manufacturing Engineering, Pusan National University, Pusan, 46241, Korea, hsm14789@naver.com

^4^ Department of Optics and Mechatronics Engineering, Pusan National University, Pusan, 46241, Korea

* Correspondence: bosung@pusan.ac.kr; Tel.: +82-51-510-2787

† These authors contributed equally to this work.

**Video S1:** Cyclic tensile strain test system composed of the Arduino railroad, computer, source meter and LCR meter.

**
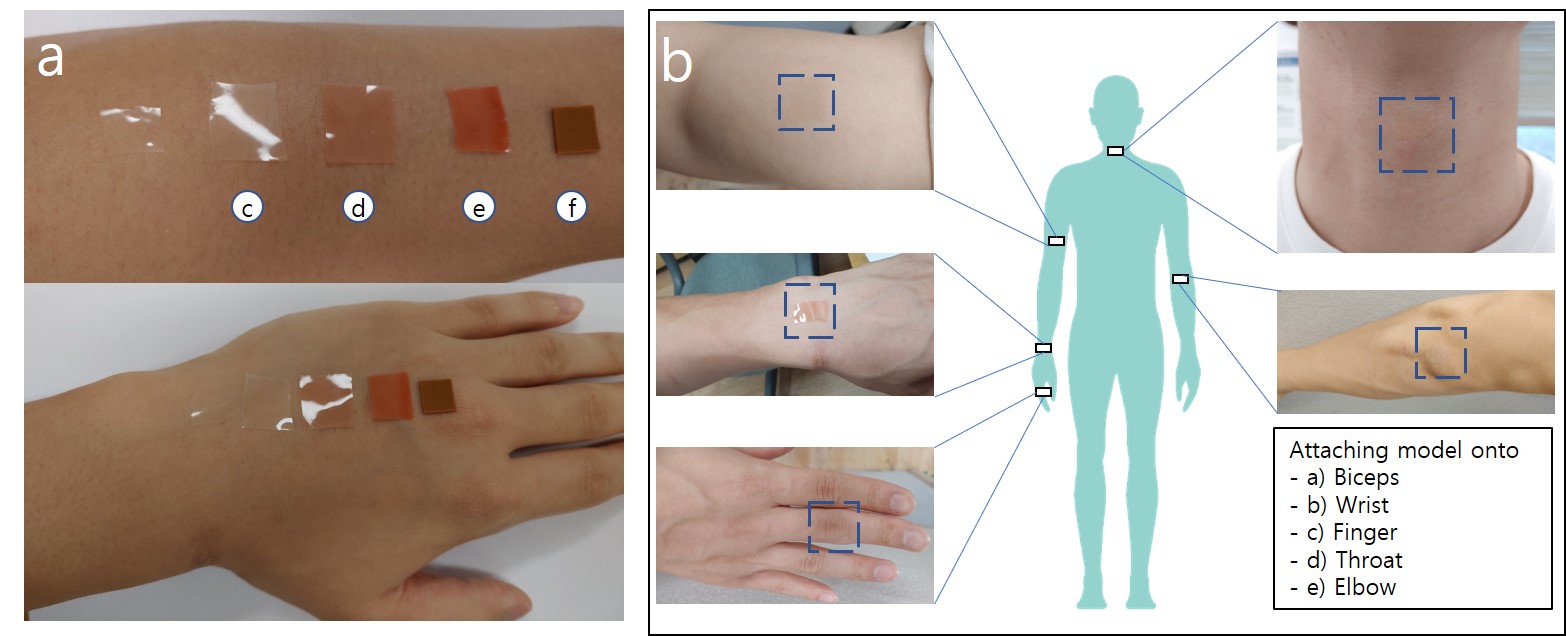
**

**Figure S1. (a)** Schematic image of the sample made through this experiment; PDMS/PSPI weight ratio for each sample; **(c)** 20:1, **(d)** 10:1, **(e)** 5:1, and **(f)** 5:2. The color of the model depends on the PSPI weight ratio. **(b)**. Schematic image of the samples attached to body parts.

**
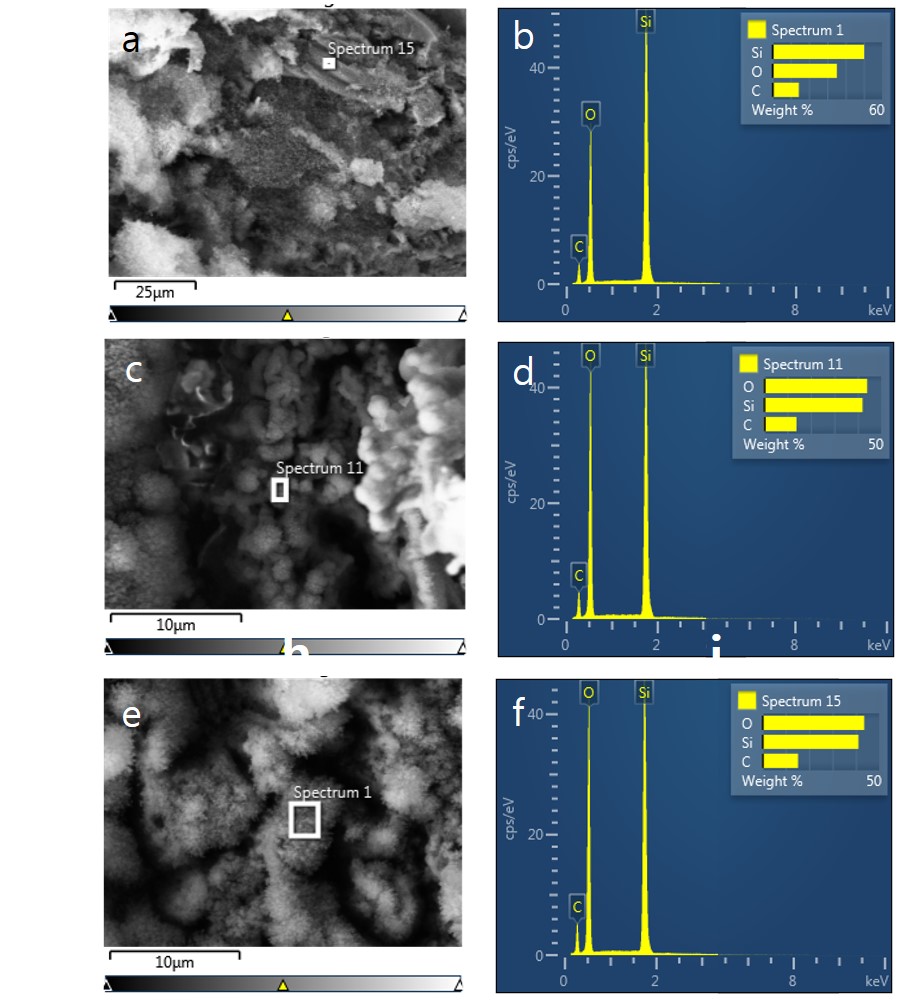
**

**Figure S2.** Elemental mapping of cotton-like materials in LIG patterns by energy dispersive x-ray (EDS) spectrometer from each sample; PDMS:PSPI by weight ratio **(a–b)** 10:1, **(c–d)** 5:1, and **(e–f)** 5:2. The smaller the weight ratio of PSPI in the model, the stronger laser power is required, which induces rapid oxygen emission in the model.

**
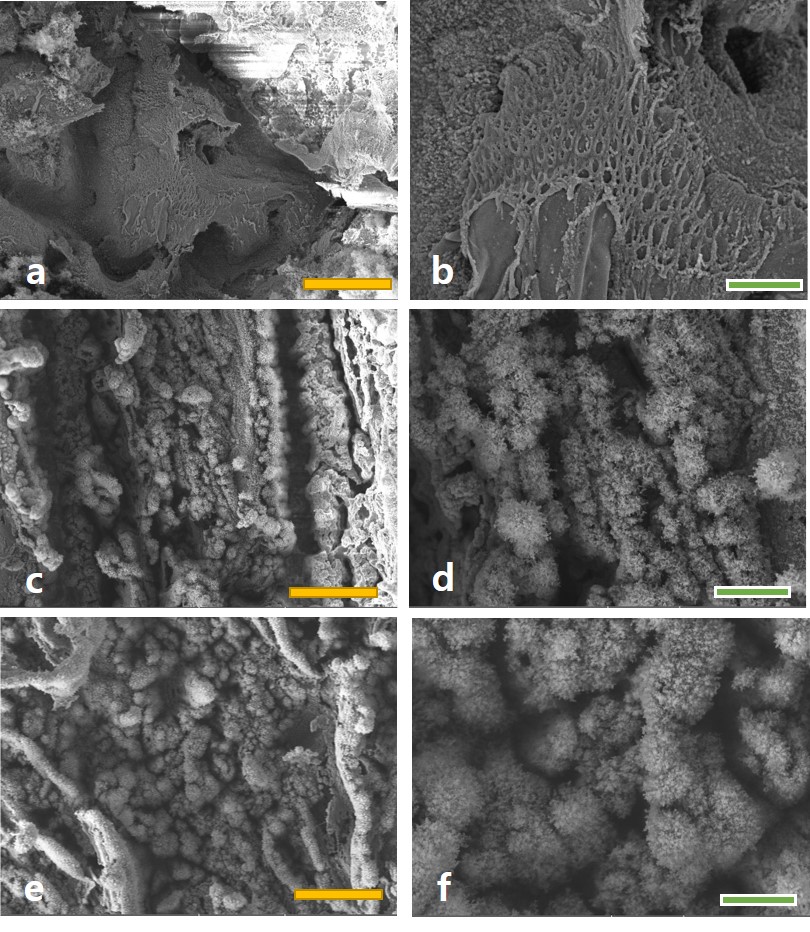
**

**Figure S3.** Top view SEM images of the skin-like electronic skin; PDMS:PSPI weight ratio; **(a)** and **(d)** 10:1, **(b)** and **(e)** 5:1, and **(c)** and **(f)** 5:2. Yellow scale bar: 20 μm and Green scale bar: 5 μm. The greater the weight ratio of PSPI in the model, the greater the proportion of cotton-like material produced per unit volume.


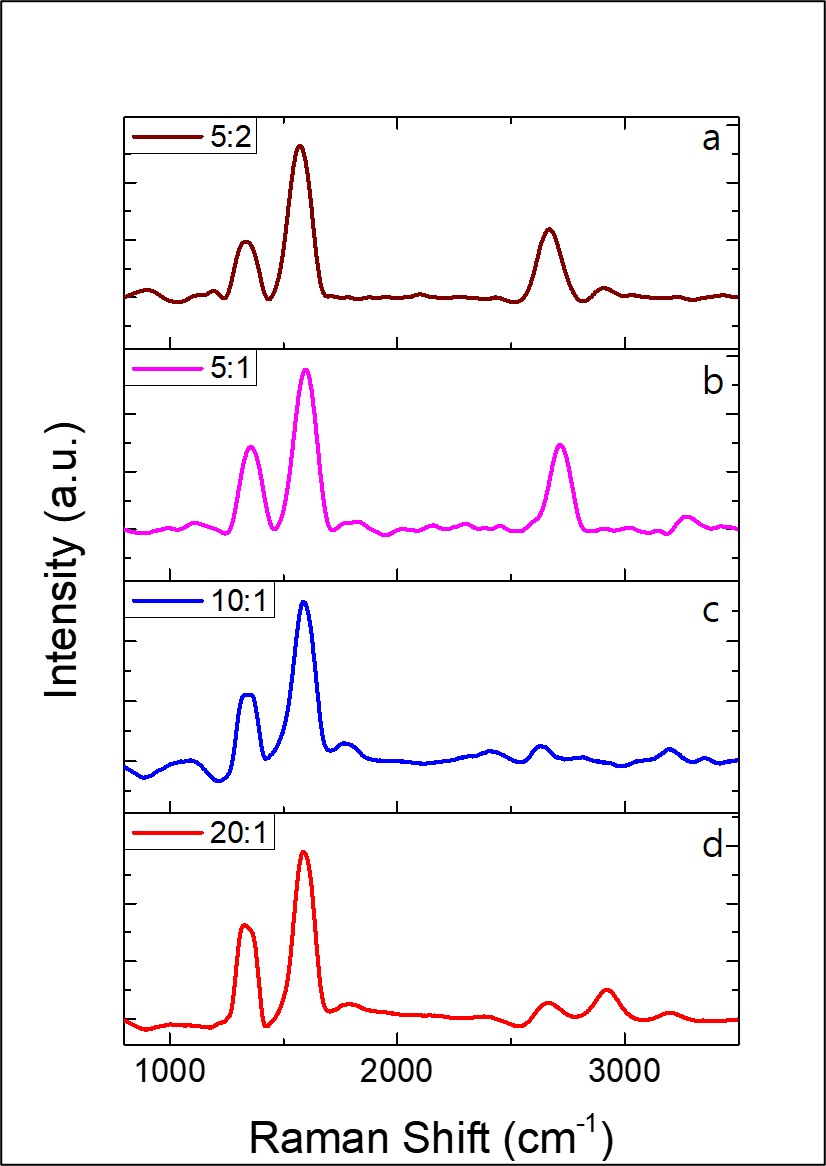


**Figure S4.** Raman spectra of controlled samples; Each skin-like polymer was carbonized by 355 pulsed laser irradiations with laser power of 1.2 W and scanning speed of 60 mm/s; skin-like polymer was fabricated by mixing two liquid-state materials (PDMS and PSPI); **(a)** 5:2, **(b)** 5:1, **(c)** 10:1, and **(d)** 20:1. Sample c and d show that these graphitic materials were similar to glassy and amorphous carbon.

**
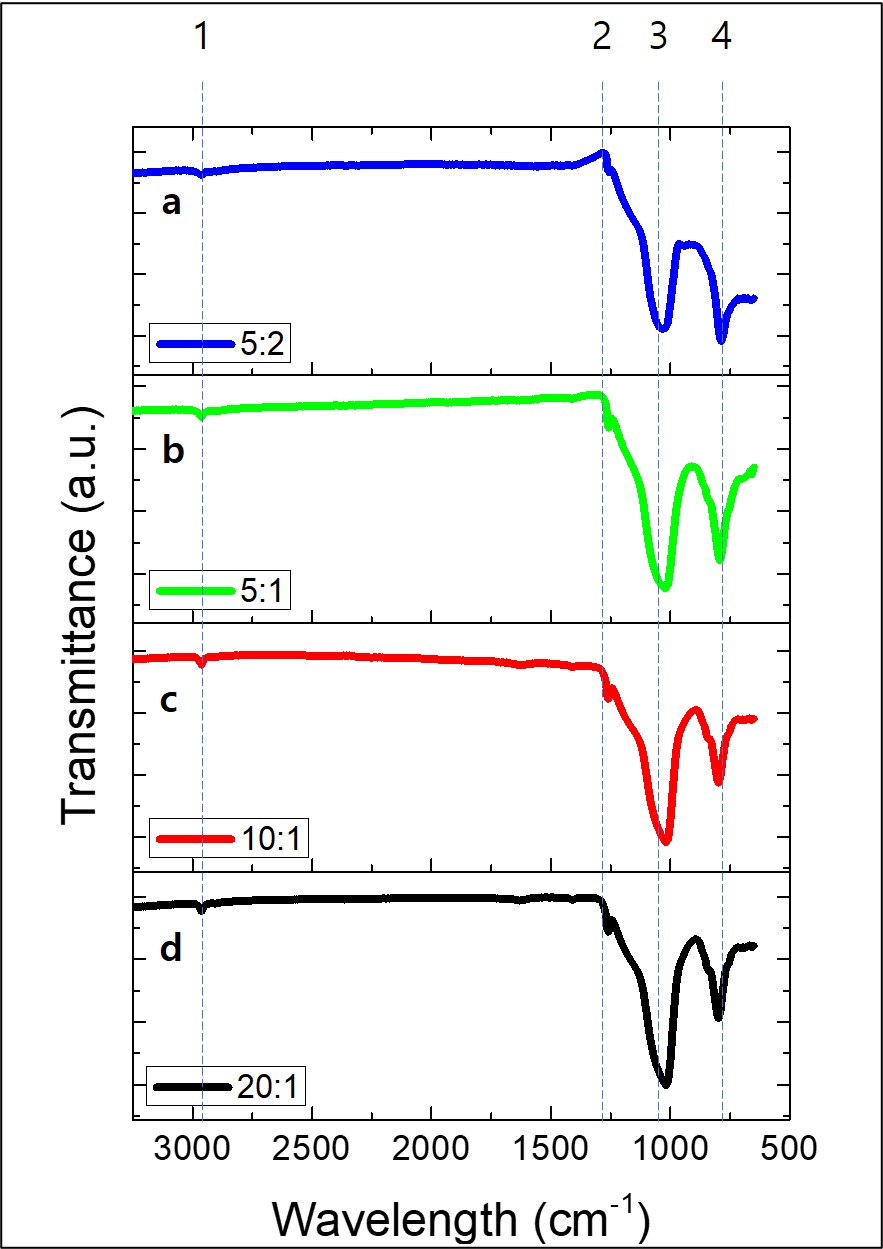
.**

**Figure S5.** FTIR spectra of e-skin fabricated with **(a)** 5:2 (PDMS:PSPI)-, **(b)** 5:1 (PDMS:PSPI)-, **(c)** 10:1 (PDMS:PSPI)-, and **(d)** 20:1 (PDMS:PSPI)- skin-like polymers. FTIR spectra of each sample show distinct transmittance in the range from 2 to 4, corresponding to the Si-C stretch, C–O–C stretching vibrations of graphene, and C-H stretching.

**
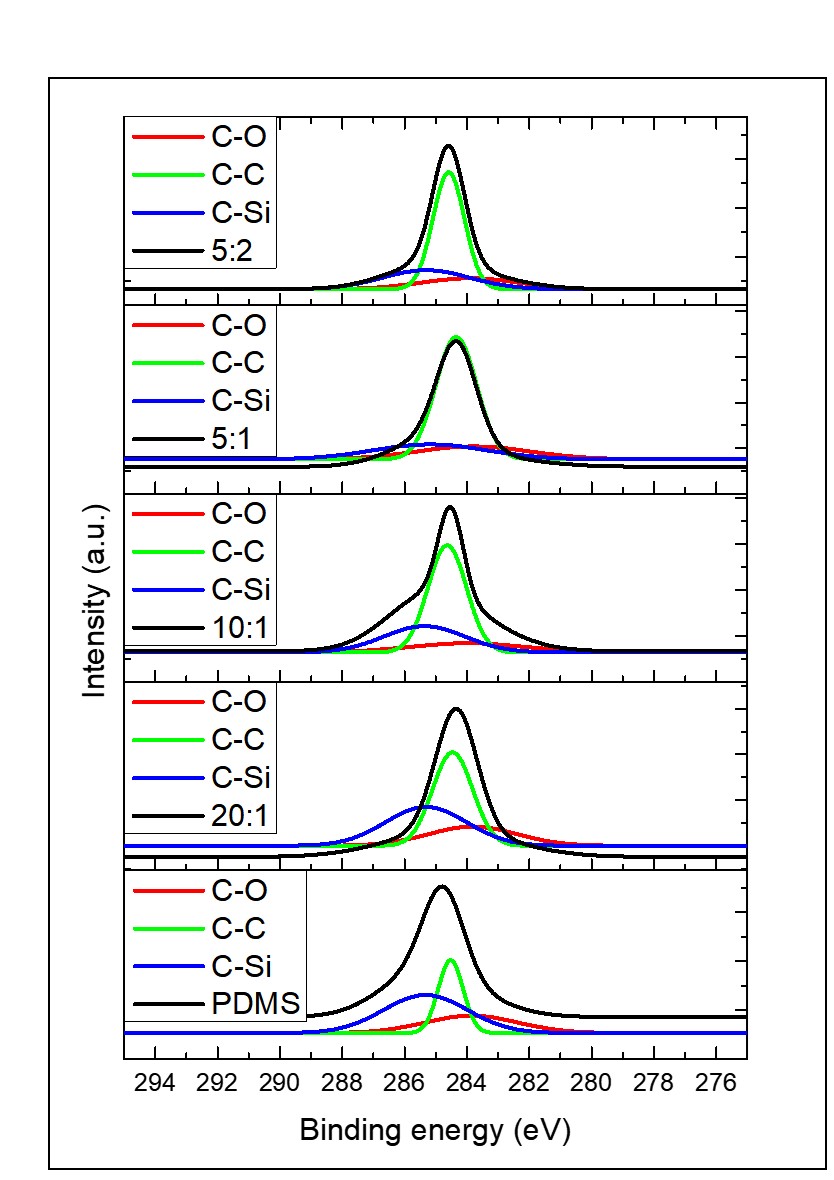
**

**Figure S6.** XPS characterization of each e-skin; In the sensing unit of e-skin fabricated after laser irradiation, as the ratio of PSPI in the polymer increases, the ratio of C-C bonds increases, and the ratio of C-Si bonds decreases.

**
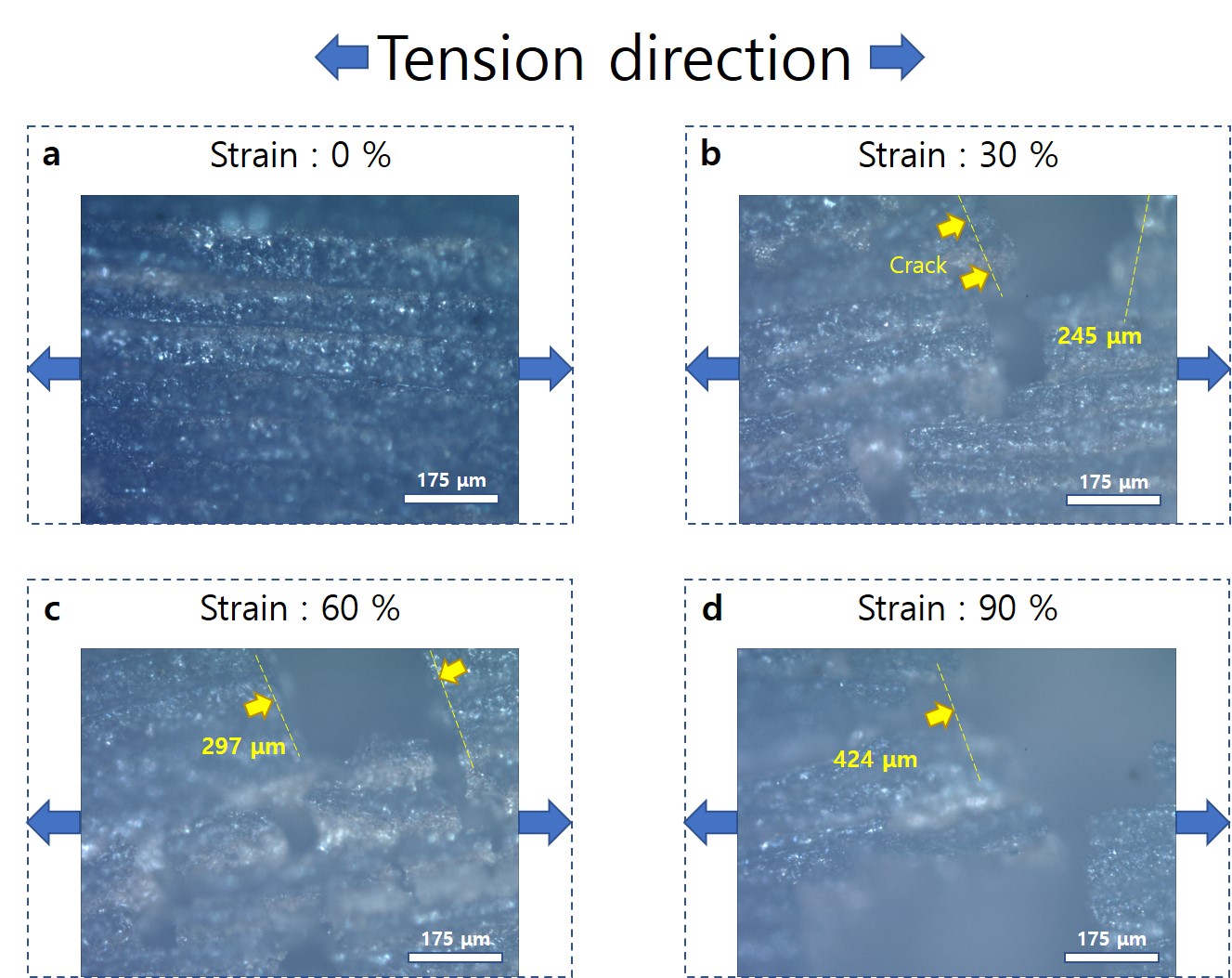
**

**Figure S7.** The Top-view of e-skin surface under strain of **(a)** 0%, **(b)** 30%, **(c)** 60%, and **(d)** 90% (Cracks generated by strain are marked as yellow). The cracks generated by the strain in the sensing unit are 245 (at 30% strain), 297 (at 60% strain), and 424 (at 90% strain) micrometers, respectively

**
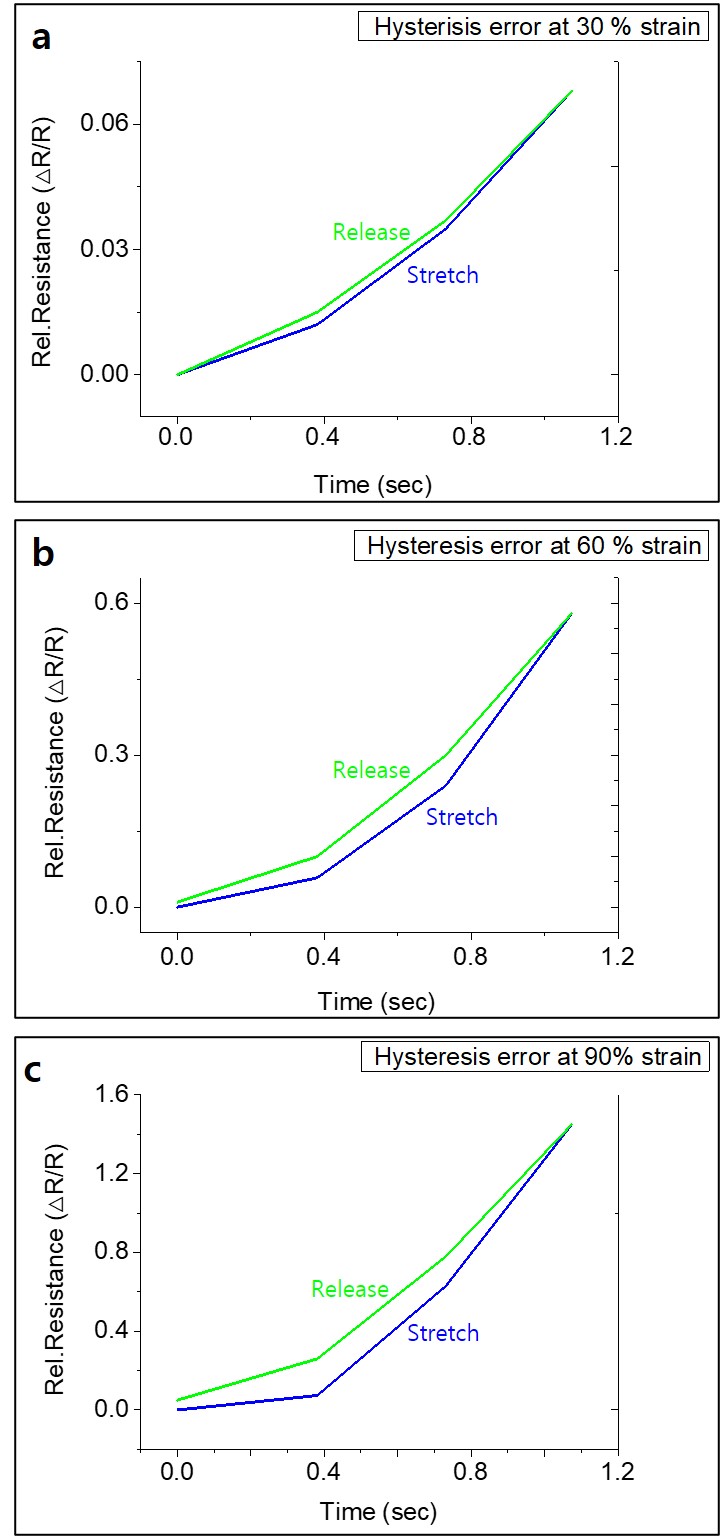
**

**Figure S8.** Hysteresis error of the E-skin under strain of **(a)** 30%, **(b)** 60% and **(c)** 90%. The e-skin showed low electrical resistance hysteresis under 30% strain and 60%.

**
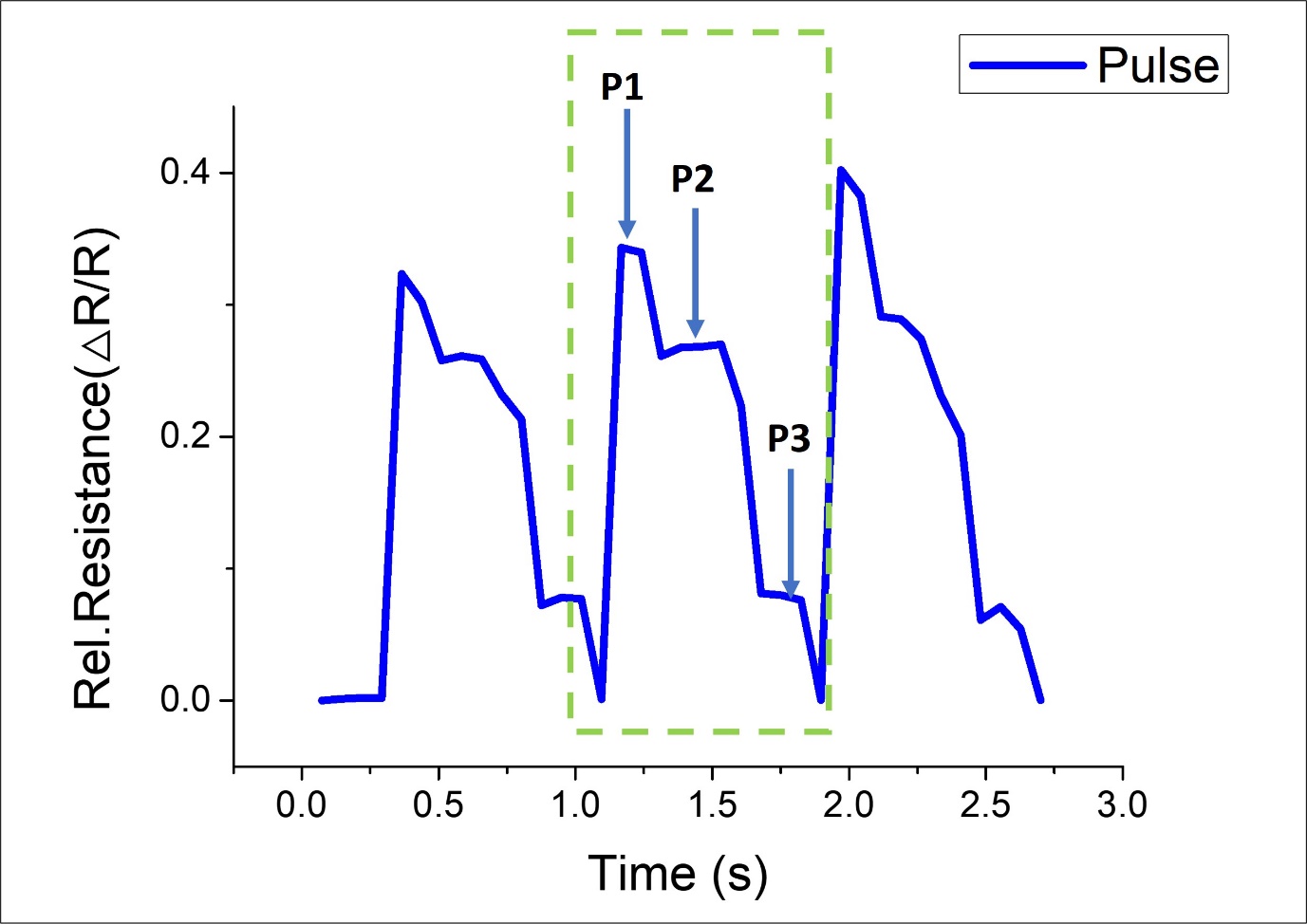
.**

**Figure S9.** The characteristic profile of wrist pulses with each composed of three peaks; P1- Percussion peak, P2- Tidal peak, and P3- Diastolic peak.
